# Effect of a printed reminder in the waiting room to turn off mobile phones during consultation: a before and after study

**DOI:** 10.1186/1471-2296-10-21

**Published:** 2009-03-23

**Authors:** Ludovic Reveiz, Sylvia de Aguiar

**Affiliations:** 1 Edificio de Consultorios Clínica Reina Sofía, Department of General Practice, Av Calle 127 # 21 – 60 cons 221, Bogota, Colombia; 2 Department of General Practice, Edificio de Consultorios Clínica Reina Sofía, Bogota, Colombia

## Abstract

**Background:**

Telephone interruptions during consultations are one cause of work-related stress amongst general practitioners. Many health care centers recommend that patients turn off any mobile phones to avoid interruptions to the discussion with the physicians.

**Methods:**

The purpose of this before and after study was to determine whether a printed reminder for turning off the mobile phone in the waiting room is helpful in decreasing the number of interruptions during consultation. A visual phone off sign utilizing the International "No" symbol of a diagonal line through a circle, along with a "please turn off your phone during consultation" reminder was used in the waiting room in the "after" period.

**Results:**

A significant difference was found in the proportion of patients receiving or making a call during the consultation (8.8% vs. 13.5%, RR = 0.66; 95%CI 0.46–0.94; p = 0.021) and in the total number of calls (10.4% vs. 17.3%, RR = 0.60; 95%CI 0.44–0.83, p = 0.003) between the exposed and the non-exposed groups. However, no significant differences were found in the total time or the median time spent talking during consultation. The duration of the calls had median times of 20.5 seconds and 22.3 seconds in the exposed and the non-exposed groups respectively. Women from both groups who received a call during consultation answered significantly more when compared to men (70% vs. 52%; p = 0.05);

**Conclusion:**

Our findings suggest that a printed reminder in the waiting room is helpful in decreasing the number of interruptions by mobile phone during consultation in our settings. The study provides the basis for further quantitative and qualitative research on this topic

## Background

Many health care centers recommend that patients turn off any mobile phones to avoid interruptions to the discussion with the doctors and nurses. In addition, many mobile phone companies and experts advise that mobiles be turned off in health care settings, as they can interfere with some critical care medical devices. Although some authors consider that fear is for the most part unjustified [1], a systematic review recommended some type of restriction of their use in hospitals, with use farther than 1 meter from electronic devices and restrictions in clinical areas [2,3]. In addition, some authors claim that the use of mobile phones should be restricted for reasons other than technical ones: "ringing tones, beeps and mobile talks may disturb other patients; ring tones could be mistaken or confused with alarms or other acoustic signals from medical equipment; sick patients may be more sensitive to noise and more easily disturbed than healthy people; calling and receiving calls may interfere with treatment; receiving calls may interfere with the tasks of the health worker"[1]. The use of mobile phones and other communication technologies is an ongoing sociocultural phenomenon that has reached into the public consciousness and is now part of "an extended humanity".

One study performed in Ireland found that 25% of general practice consultations were interrupted, and approximately 10% were significantly interrupted; over half of these interruptions were by the doctors' telephones [4]. Schattner et al found that telephone interruptions during consultations were listed as one cause of work-related stress amongst general practitioners [5].

We consider that ringing tones, beeps and mobile phone conversations may interfere with the communication between doctor and patient as it can interrupt adequate communication. In addition it may be considered discourteous to start a conversation during a medical consultation. Cell phone signs are now frequently used to gently remind patrons of proper cell phone etiquette.

The purpose of the study was to determine whether a printed reminder for turning off the mobile phone in the waiting room is helpful in decreasing the number of interruptions during consultation.

## Methods

The study had a "before" and "after" design, comparing the proportions of patients whose mobile phones rang at least once during the consultation among groups. In the "before" period, call patterns of patients – and their companion or family members – were studied under the normal operating conditions of the office. During the "after" study period a sign requesting patients to turn off the mobile phone during consultation was displayed in the waiting room. The study was implemented from February 6 to June 3, 2008 at one general practice office attending to medium and high socioeconomic status Hispanic patients in Bogotα, Colombia. Although a number of patients returned to consultation, only data from the first visit was considered. Patients with very limited vision, as reported or assessed during consultation, were also excluded from the study; all patients had the ability to read and write. A visual phone off sign (19 × 12 inches) utilizing the International "No" symbol of a diagonal line through a circle, along with a "please turn off your phone during consultation" reminder was used in the waiting room. The sign letters (black) and picture (black and red) were easily visible for all patients in the waiting room. No other signs were in the waiting room that might have caused distraction.

In the absence of previous reliable data, and based on a previous 2 weeks pilot period with 2 GPs, we estimated the proportion of ringing mobile phones during consultation to be approximately 15%. A sample size of 996 patients (498 participants in each arm) attending a general practice consultation was calculated to detect a difference of at least 35%. All consecutive participants who attended consultation were included in the study.

The primary purpose of our study was to find the proportion of patients whose mobile phones rang at least once during the consultation. In addition, we collected the following information: gender, age, whether it was the first time that the patient had attended consultation, cause of consultation (diagnosed according to the International Classification of Diseases), the average waiting time for patients in the waiting room, the proportion of patients who answered the mobile call, the length of time they spent talking on the phone, the total number of times that the phone rang, the nature of any calls made (related or not with the consultation in terms of helping to get names of medications and test results, among others) and the proportion of patients that excused themselves for receiving or making calls; we also collected information from attendants/friends/companions or family members that made calls during consultation. The length of the calls was measured using a pocket chronometer by each general practitioner when the patient responded or made a call during consultation. Both GPs were trained and standardize the way in which measurements were done. Waiting time for patients was calculated from patient registration with reception and the moment patients were seen by the general practitioner using the medical informatics systems.

### Statistical methods

The data was collected during the consultation by two physicians throughout the study in a Microsoft Access database and then transferred to SPSS version 15.0 for analysis.

Comparisons of nominal data for differences in proportions between the two study periods were performed using Pearson's chi-square (×2) test or Fisher's exact test. Differences in the means of quantitative variables were determined using the t test for independent samples. For non-normally distributed continuous data, the Mann-Whitney was used. A p-value <0.05 was required for statistical significance. We also used the Holm-Bonferroni method for multiple testing procedures [6]. This study was approved by the local research ethics committees.

## Results

The main characteristics of both groups are reported in table 1. 996 consecutive patients with a mean age of 44 years were included in the study. No significant differences were found among the exposed and the non-exposed groups regarding gender, age, whether the patient had been alone during consultation, whether it was the patient's first consultation with the physicians, average waiting time for patients in the waiting room or first cause of consultation (respiratory-related encounter which included acute respiratory infections, asthma, chronic obstructive pulmonary disease among others)* (Table 1).

**Table 1:** Characteristics of participants

	Reminder group	Non reminder group
Number of participants	498	498
Age*§	44 year (SD 15.30)	43 years (SD 14.60)
Gender*	286 (57%) women	301 (60%) women
First ever visit to the practice *	158 (32%)	143 (29%)
Average waiting time for patients in the waiting room (minutes)* §	11.71 (SD 8.46)	12.14 (8.41)
First cause of consultation (Respiratory-related encounter)*	101 (20.3%)	93 (18.7%)

*No significance; §Student's t-test

Sixty-seven patients in the non-exposed groups and forty-four patients in the exposed group received or made at least one call during consultation; a significant difference was found in the proportion of patients receiving or making a call during the consultation (8.7% vs. 13.5%, p = 0.021) and in the total number of times that the phone rang (10.4% vs. 17.3%, p = 0.002) between the exposed and the non-exposed groups (Table 2). No significant difference was found between groups in the total time spent talking (668 seconds vs. 904, p = 0.3 Mann-Whitney).

**Table 2:** Results of a printed reminder for turning off the mobile phone in the waiting room.

	Reminder group	No reminder group	RR [95%CI]	P value
Number of patients receiving or making calls	44 (8.7%)	67 (13.5%)	0.66 [0.46–0.94]	0.021
Total number of calls	52 (10.4%)	86 (17.3%)	0.60 [0.44–0.83]	0.002
Patients that answered the call,	25 (5%)	42 (8.4%)	0.57 [0.35–0.93]	0.02
Patients that excused them self for the call	7 (n = 44)	6 (n = 67)	1.17 [0.39–3.45]	0. 78

*Significant using the Holm-Bonferroni method for multiple testing procedures.

The proportion of calls answered during consultation was significantly higher in women compared (70% vs. 52% p = 0.05); however, no significant difference was found for gender when comparing the exposed and non-exposed groups. The mobile phone was useful for identifying the names and doses of medications and reading some test results that had been left at home in two and six consultations in the exposed and the non-exposed groups respectively. No call was made at the request of the doctor to get information. Finally, no significant difference was found in the number of patients making calls during consultation (0.60% vs. 1.21%, p = 0.3).

## Discussion

With this study, the authors would like to draw the attention of both patients and physicians to the fact that communication technologies are part of daily interaction in medical settings. Our study has found that having a reminder for turning off the mobile phone is helpful in decreasing the number of interruptions during consultation. The observation that almost 14% of patients take/receive cell phone calls during physician-patient interaction is surprising.

Physician-patient communication is especially important in creating a good interpersonal relationship, information exchange and improving adherence to medical recommendations and carrying out more health-related behavior changes. Patients frequently consider that communication is one of the more important competencies a physician should possess including individual behaviors and both verbal and non-verbal interactions [7-10]. A systematic review of Beck et al, explored physician verbal and non-verbal behaviors that are associated with clinical and behavioral patient outcomes in empirical studies; some of these physician behaviors were found to be positively associated with health outcomes, including empathy, courtesy and interruptions in consultation [11]. On the other hand, most physicians probably also appreciate proper cell phone etiquette from their patients. Although a reminder to turn off the cell phones is frequently used in the waiting room of consultation offices, hospitals and other health settings, a significant number of patients continue to use their phones during the medical consultation.

One study, performed in England, found that interruptions during the consultation was one of three job stressors that were predictive of high levels of job dissatisfaction for GPs [12]. In addition, rates of interruption of the consultation greatly varied from 5.3% to 95.5% and are frequently considered harmful and disruptive [13-16]. Another study found that interruptions from mobile devices were a problem, especially in surgical theatres, outpatient wards, emergency wards and inpatient rooms. The authors also proposed a system that intercept the signals from the existing communication system before they are sent out to the mobile devices and available rules defined by the doctor would decide what to do with the call [17]. The culture of the cell phone has caused changes in certain cultural norms and the ethics of this new behavior are not universally agreed upon. Cell-phone usages are frequently legitimized according to the perceived and actual "necessity," "competency," and degree of "personal freedom" [18].

There are some limitations that need to be acknowledged and addressed regarding the present study: participants were included from a unique general practice office limiting the external validity; in addition, every country and social-economic status has different mobile phone use and this may modify the number of calls received by patients. The lack of random assignment could not evenly distribute unknown confounding variables between groups, and our statistical associations do not imply causality [19]. Cultural differences may generate varied impact in doctor-patient communication and behaviors. We did not carry out a survey asking participants if they had seen the sign, how many had mobiles, or had them switched off because this may have introduced some communication bias taking into account the fact that a number of participants are related to other patients that attend the office.

Although the staff was instructed to avoid commenting on the study, the temporal effect of change due to other factors such as reception staff alerting patients or "messages" in the media could have introduced a bias. We did not include a period where the intervention was taken out to evaluate if the interruptions returned to the usual level. Although physicians were previously trained, bias may have no reliability checking of the GP measurements. Finally, we did not collect information on how often the consultation was interrupted by the physician.

## Conclusion

We have demonstrated in this preliminary study that a printed reminder in the waiting room is helpful in decreasing the number of interruptions by mobile phone during consultation in our settings. The study provides the basis for further quantitative and qualitative research on this topic. We believe that health providers should focus on reinforcing behaviors to facilitate patient-physician communication and to enhance favorable patient outcomes by using diverse strategies that may decrease interruptions during medical consultations.

## Competing interests

The authors declare that they have no competing interests.

## Authors' contributions

Both authors contributed to the protocol proposal, conducted the study and wrote the final version.

## Pre-publication history

The pre-publication history for this paper can be accessed here:


